# The betaine/GABA transporter and betaine: roles in brain, kidney, and liver

**DOI:** 10.3389/fphys.2014.00159

**Published:** 2014-04-24

**Authors:** Stephen A. Kempson, Yun Zhou, Niels C. Danbolt

**Affiliations:** ^1^Department of Cellular and Integrative Physiology, Indiana University School of MedicineIndianapolis, IN, USA; ^2^Department of Anatomy, Centre of Molecular Biology and Neuroscience, Institute of Basic Medical Sciences, University of OsloOslo, Norway

**Keywords:** synapse, leptomeninges, renal medulla, hepatocytes, osmolyte, methyl donor, mouse models

## Abstract

The physiological roles of the betaine/GABA transporter (BGT1; slc6a12) are still being debated. BGT1 is a member of the solute carrier family 6 (the neurotransmitter, sodium symporter transporter family) and mediates cellular uptake of betaine and GABA in a sodium- and chloride-dependent process. Most of the studies of BGT1 concern its function and regulation in the kidney medulla where its role is best understood. The conditions here are hostile due to hyperosmolarity and significant concentrations of NH_4_Cl and urea. To withstand the hyperosmolarity, cells trigger osmotic adaptation, involving concentration of a transcriptional factor TonEBP/NFAT5 in the nucleus, and accumulate betaine and other osmolytes. Data from renal cells in culture, primarily MDCK, revealed that transcriptional regulation of BGT1 by TonEBP/NFAT5 is relatively slow. To allow more acute control of the abundance of BGT1 protein in the plasma membrane, there is also post-translation regulation of BGT1 protein trafficking which is dependent on intracellular calcium and ATP. Further, betaine may be important in liver metabolism as a methyl donor. In fact, in the mouse the liver is the organ with the highest content of BGT1. Hepatocytes express high levels of both BGT1 and the only enzyme that can metabolize betaine, namely betaine:homocysteine –S-methyltransferase (BHMT1). The BHMT1 enzyme removes a methyl group from betaine and transfers it to homocysteine, a potential risk factor for cardiovascular disease. Finally, BGT1 has been proposed to play a role in controlling brain excitability and thereby represents a target for anticonvulsive drug development. The latter hypothesis is controversial due to very low expression levels of BGT1 relative to other GABA transporters in brain, and also the primary location of BGT1 at the surface of the brain in the leptomeninges. These issues are discussed in detail.

## Background

Osmotic stress occurs in several tissues and has been studied most extensively in cells in the inner medulla of the kidney. In humans these cells are normally exposed to low oxygen tension, ammonia, and very high levels (600 mOsm) of both NaCl and urea when urine is maximally concentrated. Numerous perturbations can result from the hypertonic effect of high NaCl and the denaturing effect of high urea. These include production of reactive oxygen species, cytoskeletal rearrangements, inhibition of DNA replication, transcription, and translation, and damage to DNA and mitochondria (Burg et al., 2007; Cheung and Ko, 2013). Adaptation is essential for survival and the adaptations by medullary cells are extensive (Lee et al., 2011). Many are driven by the transcription factor TonEBP/NFAT5 (Burg et al., 1997; Cheung and Ko, 2013). Compared to cells grown in culture, renal medullary cells appear to be more tolerant *in vivo* in part because conditions change more slowly and because adaptation to osmotic stress may confer tolerance to other stresses (Santos et al., 2003). However, cell death occurs by apoptosis when the adaptations fail (Go et al., 2004; Lam et al., 2004; Lopez-Rodriguez et al., 2004; Moeckel, 2013). The adaptive mechanisms include increased expression of heat shock proteins and accumulation of organic osmolytes (Neuhofer and Beck, 2005; Kwon et al., 2009). These osmolytes are termed “compatible” because, in contrast to electrolytes, they do not perturb the function of macromolecules when present at high intracellular concentrations (Yancey et al., 1982).

Betaine, which is found in many foods including spinach and wheat, is also one of the important osmolytes in the kidney medulla. Betaine transport activity was discovered in Madin-Darby canine kidney (MDCK) cells (Nakanishi et al., 1990), and screening of a MDCK cell cDNA library for expression of betaine transport activity in *Xenopus* oocytes resulted in isolation of a betaine transporter cDNA (Yamauchi et al., 1992). The nucleotide sequence turned out to be closely related to those of brain transporters for γ-amino-*n*-butyric acid (GABA) and noradrenalin (Gadea and López-Colome, 2001; Eulenburg and Gomeza, 2010). Because the novel transporter was able to transport not only GABA, but also betaine, it was named the betaine-GABA transporter (BGT1; slc6a12). In parallel, two other research groups independently cloned BGT1 homologs from mouse (López-Corcuera et al., 1992) and human brain (Borden et al., 1995a) based on homology to GABA transporter 1 (GAT1; slc6a1) (Guastella et al., 1990). Soon thereafter, BGT1 was also cloned from rat liver (Burnham et al., 1996) and from human kidney (Rasola et al., 1995). Most of the studies of BGT1 concern its function and regulation in the kidney medulla where its role in osmolyte transport has been well-defined. Its presence in the brain and liver is well-documented, but its role in these tissues has not been studied extensively.

It is important to note that the mouse isoform was originally called mouse GAT2 or mGAT2 (López-Corcuera et al., 1992) and that BGT1 therefore should not be confused with rat/human GAT2 (slc6a13) which does not transport betaine. BGT1 expression may vary among species. In the dog, BGT1 mRNA was primarily detected in the kidney while the levels in the brain and liver were considerably lower (Roberts, 1974; Yamauchi et al., 1992). The highest BGT1 mRNA levels in the mouse were found in the liver (López-Corcuera et al., 1992) while the highest levels in man were in the kidney followed by the liver (Rasola et al., 1995).

## BGT1 and betaine in brain

Because GABA is the major inhibitory neurotransmitter in the adult mammalian brain (Roberts, 1974; Krnjevic, 2004) and its action is terminated by cellular uptake catalyzed by the GABA transporters, these transporters are highly interesting as targets for the development of anticonvulsant and antiepileptic drugs (Gadea and López-Colome, 2001; Eulenburg and Gomeza, 2010). The mammalian genome contains four genes encoding GABA transporters, namely GAT1 (slc6a1), GAT2 (slc6a13), GAT3 (slc6a11), and BGT1 (slc6a12). Tiagabine (marketed under the name Gabitril®) is the only clinically approved GABA transporter inhibitor, and is used for treatment of mesial temporal lobe epilepsy. The use of tiagabine, however, is limited by its short half-life and side effects (dizziness, fatigue, confusion, tremor, ataxia, and nervousness). Side effects are not surprising considering that tiagabine is a selective GAT1 inhibitor (Nielsen et al., 1991; Borden, 1996; Krogsgaard-Larsen et al., 2000) and that GAT1-knockout mice display similar signs (Chiu et al., 2005). On this background it makes sense to determine if the other GABA transporter subtypes (GAT2, GAT3, and BGT1) represent better drug targets. Consequently, medicinal chemists developed a number GABA uptake inhibitors (Krogsgaard-Larsen et al., 2000; Soudijn and van Wijngaarden, 2000; Andersen et al., 2001) as well as novel assays for compound screening (Sindelar and Wanner, 2012; Polley et al., 2013).

On this background, a novel GABA uptake inhibitor *N*-[4,4-bis(3-methyl-2-thienyl)-3-butenyl]-4-(methylamino-4,5,6,7-tetrahydrobenzo[d]isoxazol-3-ol (EF1502), which inhibits both GAT1 and BGT1, but not GAT2 and GAT3, was produced (Clausen et al., 2006). This compound was subsequently reported to be synergistic to the GAT1 inhibitor tiagabine in protection against seizures. These observations were interpreted as evidence for a functional role for BGT1 in seizure control (White et al., 2005; Clausen et al., 2006), and investigations of brain BGT1 suddenly became a hot topic.

However, a role of BGT1 in the brain was surprising considering the low expression levels (López-Corcuera et al., 1992; Burnham et al., 1996) and lower affinity for GABA than those of the other GABA transporters. The reported Km values for the mouse isoforms are 0.8, 8, 18, and 80 μM, respectively, for GAT3, GAT1, GAT2, and BGT1 (López-Corcuera et al., 1992; Liu et al., 1993; Matskevitch et al., 1999). This was clear to Borden (Borden, 1996) and he wrote that BGT1: “is unlikely to play a major role in terminating the action of GABA at a synapse” due to low levels. But then, possibly to keep all options open, added that: “It may, however, serve to sequester GABA that has diffused away from synaptic regions, thereby assuring the fidelity of transmission.” [This argument seemed plausible based on the limited understanding of neurotransmitter diffusion at the time, but see below]. In agreement, Smith et al. (2008) reported that they had used two GABA transport inhibitors to modulate inhibitory tone via inhibition of GAT1 (with tiagabine) or BGT1 (with EF1502) and found differential effects in an *in vitro* model of spontaneous interictal-like bursting. This paper also cites a paper by Ahn et al. (1996) for support to the notion that BGT1 is in dendrites. However, in this study BGT1 cDNA was microinjected into cultured hippocampal neurons. When that was done, it was found that BGT1 was primarily targeted to the dendrites, but (obviously) that does not tell if cells in the brain actually express the protein in the first place.

Researchers tried to localize BGT1 in the brain and in cell cultures (Borden, 1996), but there was a great deal of uncertainty. BGT1 mRNA was reported in cultured astrocytes and in an astrocytoma cell line, but not in cultured neurons (Borden et al., 1995b; Bitoun and Tappaz, 2000; Ruiz-Tachiquin et al., 2002). BGT1 protein was reported in brain endothelium (Takanaga et al., 2001), in astrocyte and astrocytoma cultures, under hyperosmotic conditions in particular (Ruiz-Tachiquin et al., 2002; Olsen et al., 2005), in pyramidal neurons (but not astrocytes) in untreated rats (Zhu and Ong, 2004a), in astrocytes in kainate injected rats (Zhu and Ong, 2004a) and in *Macaca fascicularis* monkeys (Zhu and Ong, 2004b). The latter investigators observed BGT1 label in dendritic spines, not at GABAergic synapses, but at glutamatergic synapses and referred to this as BGT1 being localized in “an extra-perisynaptic region, away from the post-synaptic density” (Zhu and Ong, 2004b). This was interpreted as evidence in support of Borden's suggestion (see above). However, these immunocytochemical data could not be validated because knockout animals were unavailable at the time to serve as negative controls. For the importance of this see our previous studies (Holmseth et al., 2006, 2012). Further, these data were mostly based on the same antibody from Chemicon (Temecula, CA, USA) to the 15 C-terminal amino acids of rat BGT1. Unfortunately, we now know that this sequence differs between species raising concerns about the specificity:

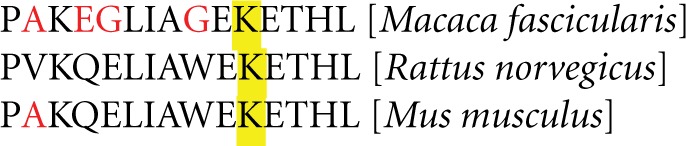



It is still possible that the antibodies recognize the monkey version, but according to our experience with peptide antibodies this is not very likely (Danbolt et al., 1998; Holmseth et al., 2005; Holmseth et al., 2012) and it was not tested (Zhu and Ong, 2004a,b) whether the antibody recognized after aldehyde fixation (Holmseth et al., 2012) as a lysine residue (yellow) can be affected by fixation. Further, there is poor correlation between the brain regions reported to have the highest immunoreactivity (Zhu and Ong, 2004a,b) and those with the highest BGT1 mRNA levels (Zhou et al., 2012a). Thus, these immunocytochemistry data can be disregarded. Having said that, our data (Zhou et al., 2012a) do not exclude the possibility that the labeling observed in astrocytes after kainate injection could be due to BGT1 (Zhu and Ong, 2004a,b). But if BGT1 is present in astrocytes, then it will be expressed in between a much higher number of GAT3 transporters (Conti et al., 2004) which have (as explained above) also higher affinity. This would in itself invalidate the notion about “an extra-perisynaptic region away from the post-synaptic density.”

This led to a great deal of uncertainty and, at the same time, the questions were important. If EF1502, despite low BGT1 expression levels, did mediate its effects via BGT1 then there might be something new and important to learn. Alternatively, if EF1502 did not mediate its effects via BGT1 then it might be worthwhile to identify the other target. To resolve this issue, BGT1 knockout mice were developed (Lehre et al., 2011) and subjected to seizure threshold testing. To exclude differences caused by other factors, such as age, gender, and environment, BGT1 wild type and knockout littermates were used. The mice were subjected to a series of seizure threshold tests including pentylentetrazol (PTZ) seizure threshold test, minimal clonic seizure threshold test, the 6 Hz seizure threshold tests, and minimal tonic extension threshold test (Lehre et al., 2011). Unexpectedly, no differences in seizure thresholds were noted between BGT1 knockout and wild type mice (Lehre et al., 2011). EF1502 is an anticonvulsant in both the BGT1 wild type and knockout mice presumably because of its dual function of also inhibiting GAT1. In fact, in our hands, EF1502 was somewhat more potent in the BGT1-deficient mice (Lehre et al., 2011). Thus, these experiments did not provide any indication whatsoever that BGT1 plays a role in controlling seizure thresholds.

**Figure 1:**
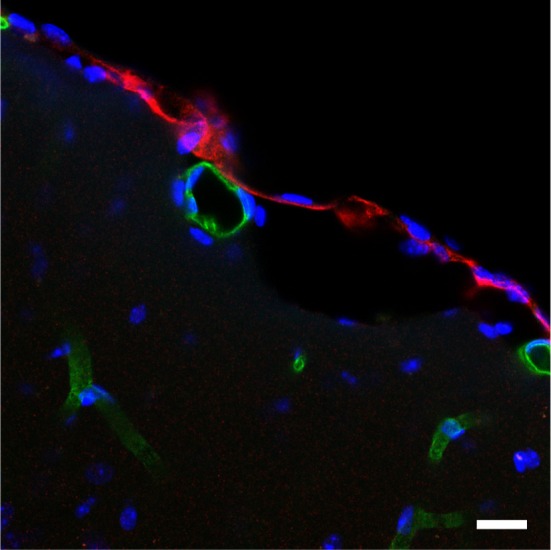
**BGT1 is expressed in the leptomeninges**. The sections from wildtype and knockout were labeled with anti-BGT1 antibody (red; Ab#590; 1 μg/ml), and with anti-CD31 antibodies (green; 0.5 μg/ml; endothelial marker). The images from the sections from knockout mice are not shown here. Scale bars = 20 μm. Immunochemistry was performed using the same materials and procedures described in detail by Zhou et al. (2012a).

Lack of differences in seizure thresholds needed an explanation, and the BGT1 expression levels and distributions were determined using the knockout mice as negative controls to verify labeling specificity. Although it was already known that the brain BGT1 levels were low, it was unexpected to find that BGT1 is expressed at levels 2–3 orders of magnitude lower than those of GAT1 in mice (Lehre et al., 2011) and that most of BGT1 is located at the surface of the brain, in the leptomeninges, rather than in brain tissue proper (Figure 1), also see Figure 9 in Zhou et al. (2012a). This result is further supported by an *in situ* hybrization study (Evans et al., 1996) and mass spectroscopy data (Nielsen et al., 2005; Lu et al., 2009; Walther and Mann, 2011). As GAT2 is also expressed at low levels in mouse brain tissue proper (Zhou et al., 2012b), this implies that GAT1 and GAT3 are the most important ones in the rodent central nervous system (Conti et al., 2004; Zhou and Danbolt, 2013).

In quiescent tissue the ambient GABA levels around synapses are relatively low (Westergren et al., 1994), possibly as low as suggested for glutamate (Herman and Jahr, 2007), and thereby well-below the Km of GAT3. The mouse isoforms of GAT1 and GAT3 have, respectively, about 10 and 100 times higher affinity for GABA than BGT1. Thus, GABA is assumed to be effectively removed to a level where BGT1 becomes inefficient. Because GABA levels can rise to high levels during periods of intense synaptic activity (Semyanov et al., 2004; Olah et al., 2009), it is conceivable that there are situations when BGT1 may be activated. However, this is highly unlikely to have any effect on GABA uptake because our comparison indicates that the BGT1 levels are approximately 100–1000 times lower than those of GAT1, and available data from the literature (Mager et al., 1993; Sacher et al., 2002; Karakossian et al., 2005; Gonzales et al., 2007) do not suggest that BGT1 is any faster than the other GABA transporters. Thus, even when GABA levels are high, the BGT1 can at most be responsible for 0.1–1.0% of the transport. The contribution will be far less at lower GABA levels. Furthermore, neurotransmitters diffuse rapidly out of the synaptic cleft on a low microsecond timescale until they bind to transporters and are removed (Clements, 1996; Danbolt, 2001; Lehre and Rusakov, 2002; Rusakov et al., 2005). Thus, unless there are more transporters in the immediate vicinity of synapses than released GABA molecules, then GABA molecules will pass by all occupied transporters diffusing multiple synapse diameters away before the occupied transporters are ready for a new transport cycle. This rules out a role for BGT1 in controlling GABA levels in mice, and explains why there were no significant differences between wildtype and BGT1-deficient mice in seizure thresholds. Thus, if BGT1 plays any role in brain tissue proper this cannot be due to its ability to transport GABA. Its role must be either to transport something else or a novel function requiring very few BGT1 molecules.

Recently a novel role of BGT1 and betaine has been proposed (Peden et al., 2013) based on studies in *C. elegans*. The betaine transporter in this nematode species is termed SNF-3 and is a BGT1 ortholog that is Na^+^ and Cl^−^ dependent. However, SNF-3 differs from mammalian BGT1 in that it transports only betaine not GABA. The SNF-3 functions primarily in the epidermis where its role is to remove extracellular betaine which is toxic to nematodes. The toxicity is probably due to constitutive activation of the betaine receptor by excess extracellular betaine. The receptor is a betaine gated cation channel (ACR-23) which is localized in body muscle and neurons (Rufener et al., 2013). Inactivation of SNF-3 was associated with hypercontraction and paralysis of the worms due to excess betaine (Peden et al., 2013). This study suggests that betaine has a signaling role and some earlier studies in mice lend support to this notion. For example, betaine was reported to have anti-epileptic properties (Freed et al., 1979), and can partly rescue the brain atrophy in MTHFR deficient mice (Schwahn et al., 2004). More recently it was reported that betaine can elevate growth hormone levels and activate IGF-1 signaling pathways in pig, mouse and rat tissues (Lee et al., 2006; Huang et al., 2007; Senesi et al., 2013).

The vertebrate brain, however, contains low amounts of betaine (Gullans and Verbalis, 1993; Slow et al., 2009) (Table 1) which is probably derived from exogenous betaine (Schwahn et al., 2004) via non-specific organic solute transporters across the blood brain barrier. Most of the dietary betaine is utilized by liver and kidney (Pummer et al., 2000; Kettunen et al., 2001; Kim et al., 2003), see detailed discussion below. Further, the need for high doses (intraperitoneal) to attenuate PTZ-induced seizures may be due to poor transport across the blood brain barrier because betaine was more potent when injected directly into the ventricles (Ghoz and Freed, 1985). However, as explained above BGT1 protein is, at least in mice, not significantly present in either neurons or glia (Zhou et al., 2012a) implying that the betaine removal capacity around synapses is extremely low. This is also consistent with the finding that wildtype and BGT1-deficient mice have similar seizure thresholds (Lehre et al., 2011). However, considering that BGT1 is expressed in the leptomeninges (Zhou et al., 2012a), it is legitimate to ask if BGT1 can contribute to betaine removal via the newly discovered glymphatic pathway, which is a key contributor to the clearance of interstitial solutes (Iliff et al., 2012). Further studies are needed before any firm conclusion can be made concerning a signaling role of betaine and BGT1 in the mammalian brain.

**Table 1:** **Summary of osmolyte concentrations in mouse tissues**.

**Content**	**Brain (μmol/g)**	**Kidney (μmol/g)**	**Liver (μmol/g)**	**References**
Betaine	0.02–0.1	1–5	1–10	Schwahn et al., 2004; Clow et al., 2008; Teng et al., 2011
Taurine	5–10	8–20	5–15	Brosnan and Brosnan, 2006; Warskulat et al., 2006; Zhou et al., 2012b; Roman et al., 2013; Bjørnsen et al., 2014
*myo*-inositol	5–7	6–7	0.3–0.6	Chau et al., 2005; Bjørnsen et al., 2014

Could BGT1 and betaine play a role in modulating cell volume in the brain? Considering the abundance of the other osmolyte transporters SMIT and TAUT and the relative levels of osmolytes in the brain (Table 1) (Heilig et al., 1989; Zhou et al., 2012a) a role of BGT1 in maintaining cellular volume in brain is probably insignificant. This argument is further strengthened by the low level of brain betaine (Slow et al., 2009), the lack of change of brain betaine content with either salt loading or water deprivation (Heilig et al., 1989; Lien et al., 1990; Zhou et al., 2012a), and the lack of a significant increase of BGT1 mRNA in brain during acute and chronic salt loading (Kaneko et al., 1997).

In summary the roles of BGT1 and betaine in brain, and possible regulation by TonEBP/NFAT5, have not been determined. As discussed above, BGT1 is unlikely to be involved in GABA and betaine transport in brain tissue proper, at least in normal mice brains. Thus, the hypothesis reviewed in Madsen et al. (2010) that inhibition of BGT1 can delay removal of GABA from extrasynaptic GABA receptors due to its “peri-extrasynaptic localization” is refuted. On the other hand, the fairly high levels of BGT1 in the leptomeninges suggest that BGT1 is present in sufficient concentrations to perform a physiologically relevant function although the nature of this function remains to be discovered.

## BGT1 and betaine in kidney

It is now well-established that the primary role of betaine in the kidney is osmoprotection. The always high but changing extracellular osmolarity in the kidney medulla plays an essential role in urine concentration. To balance the high osmolarity and preserve cell volume without interfering with cell function, one well-characterized mechanism is to accumulate compatible osmolytes (Miyakawa et al., 1999a). Betaine, sorbitol, myo-inositol, taurine, and glycerolphosphorylcholine are the predominant osmolytes in the mammalian kidney and MDCK cells (Bagnasco et al., 1986), and the function of these osmolytes had been discussed in detail elsewhere (Burg and Ferraris, 2008). The accumulation of betaine is primarily due to the presence of BGT1 and is influenced by tonicity (Nakanishi et al., 1990; Yamauchi et al., 1992; Handler and Kwon, 1996). MDCK cells have been used extensively as a model for studying the potential functions of BGT1 in the kidney, in part because they were derived from the distal nephron (Ojakian et al., 1987) and because a wealth of information on epithelial cell behavior is available from studies in this cell line (Dukes et al., 2011). Under the isotonic condition BGT1 is mainly in the cytoplasm in MDCK cells (Basham et al., 2001; Kempson et al., 2003) and the betaine transport capacity of the cells is low (Nakanishi et al., 1990; Kempson, 1998). Hypertonicity increases betaine transport, primarily in the basolateral plasma membrane, by activating transcription of the BGT1 gene. This increases abundance of BGT1 mRNA more than 10-fold and increases the transport capacity 5–10 fold (Yamauchi et al., 1991; Uchida et al., 1993; Kempson, 1998). Activation of transcription is achieved by tonicity-responsive enhancer sequences (TonE1 and TonE2) that are present in the promoter region of the BGT1 gene and specifically bind the TonE binding proteins (TonEBP/NFAT5's) (Takenaka et al., 1994; Miyakawa et al., 1998, 1999b). Up- and down-regulation of BGT1 mRNA transcription in response to changes in medullary tonicity has been confirmed also *in vivo* (Kaneko et al., 1997). In MDCK cells the transcriptional regulation of BGT1 by TonEBP/NFAT5 and subsequent upregulation of betaine transport is relatively slow, requiring up to 20 h for completion (Yamauchi et al., 1991; Kempson, 1998). Using the same cell line we have identified more acute control of plasma membrane abundance of BGT1 (Kempson et al., 2003, 2005; Kempson and Montrose, 2004; Lammers et al., 2005). In addition we have reported changes in post-translational regulation of BGT1 protein trafficking within 30–60 min in response to changes in concentrations of extracellular calcium (Kempson et al., 2006; Parikh et al., 2013), ATP and adenosine (Kempson et al., 2008). The common mechanism is endocytic removal of pre-existing BGT1 protein from the basolateral plasma membrane which would be useful when cellular accumulation of betaine was no longer required. In contrast, nitric oxide which is produced in the renal medulla is response to hypertonic extracellular NaCl (Kempson et al., 2007) upregulates BGT1 transport in MDCK cells. The mechanism is not understood but the result is increased delivery of BGT1 protein to the plasma membrane, as detected by *total internal reflection fluorescence* (TIRF) microscopy (Kempson et al., 2011). The response to nitric oxide may be complex since an earlier study reported that nitric oxide directly inhibits the transcriptional activity of TonEBP/NFAT5 (Neuhofer et al., 2009). It is notable that BGT1, unlike the other GABA transporter subtypes, is acutely regulated by extracellular pH (Matskevitch et al., 2000; Grossman and Nelson, 2002, 2003). In summary, these acute acting factors provide a system of checks and balances that fine-tune the capacity for betaine accumulation during fluctuations in hypertonicity in the renal medulla.

At the molecular level, the transport mechanism and specific sites that bind and couple betaine transport to the movement of Na^+^ and Cl^−^ ions are not understood. This is in marked contrast to the detailed information on bacterial Na^+^-coupled betaine transporters such as BetP (Ott et al., 2008; Perez et al., 2012). The basolateral targeting information for BGT1 lies within a short segment of amino acids (565–572) that is rich in basic residues and located within the cytoplasmic C-terminus (Perego et al., 1997). An additional requirement for accurate targeting during hypertonic upregulation appears to be phosphorylation at T40 located in a cytoplasmic loop near the N-terminus. This is based on our observation that when T40, a potential phosphorylation site, was mutated to alanine to prevent phosphorylation the T40A mutant form of BGT1 was trapped intracellularly in the trans-Golgi network. In contrast, when T40 was mutated to either glutamate or aspartate to mimic phosphorylation at T40, both the T40E and T40D forms of BGT1 were found to traffic normally to the plasma membrane during hypertonic stress (Anderson et al., 2010; Day et al., 2012) (Kempson, unpublished data). This suggests that T40 near the N terminus may be part of a hot spot for normal trafficking or insertion of BGT1 in the plasma membrane. Following delivery and insertion, the retention of BGT1 in the plasma membrane depends on the association between BGT1 and the LIN7 PDZ membrane protein which involves a PDZ target sequence in the last 5 residues at the C terminus of the transporter. This association is regulated by protein kinase C and T612 is an essential target. Phosphorylation of T612 disrupts the association and leads to internalization of BGT1 (Massari et al., 2005). Apart from these few studies, little is known about structure-function relationships in renal BGT1.

**Figure 2:**
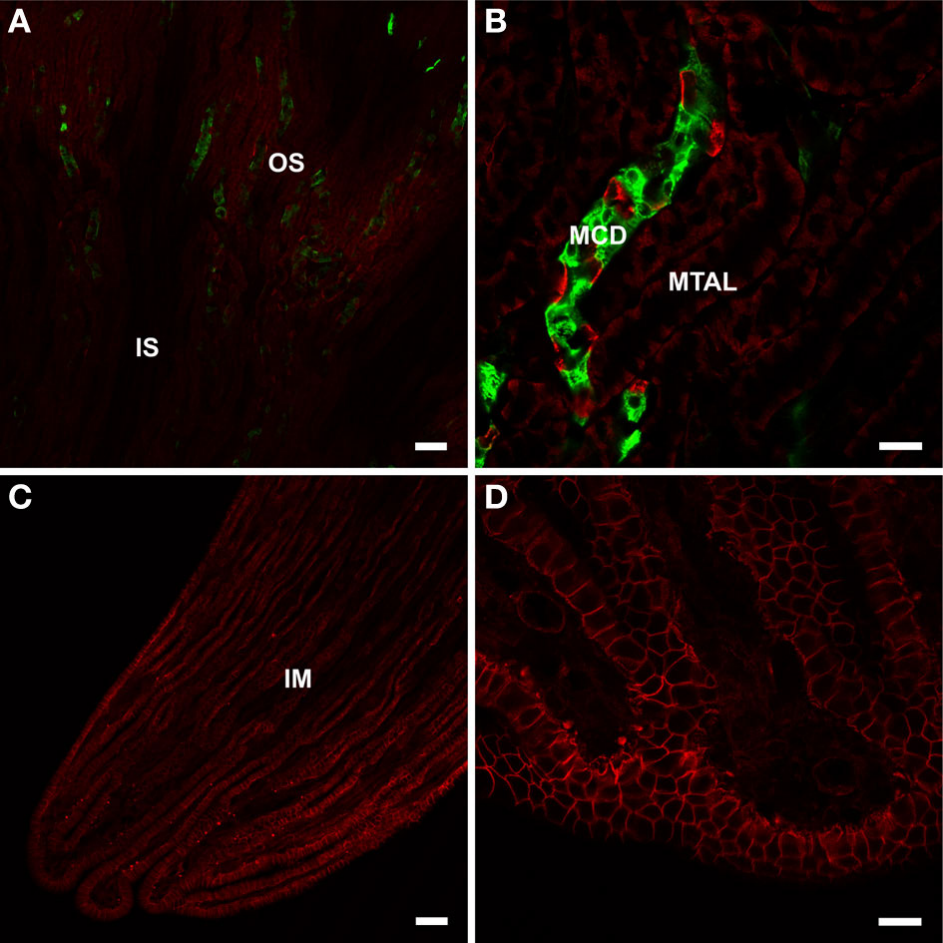
**BGT1 localization in the kidney**. The kidney sections from wildtype and knockout were labeled with anti-BGT1 antibody (red; Ab#590; 1 μg/ml), and with fluorescein-conjugated *D. biflorus* agglutinin (green; 1:300; marker for collecting ducts). **(A,B)** are from the outer strip of outer medulla, and **(C,D)** are from the tip of the renal papilla. The images from the sections from knockout mice are not shown here. Scale bars for **(A,C)** = 50 μm; scale bars for **(B,D)** = 20 μm. Immunochemistry was performed using the same materials and procedures described in detail by Zhou et al. (2012a).

Even though BGT1 has been suggested for more than two decades to play an important role in osmolyte accumulation and survival of the renal medullary cells, the localization of BGT1 protein *in vivo* was not reported until recently. Zhou and colleagues detected BGT1 protein at low levels in the renal outer medulla, and at very high concentration in the renal papilla (Figures 2A,C). Also see Figure 6 in Zhou et al. (2012a) which is in agreement with the *in situ* hybridization data (Miyai et al., 1996). BGT1 protein was in the basolateral membrane of cells in the collecting ducts, and in the cytoplasm of cells in the thick ascending limb of Henle's loop. Also see Figure 7 in Zhou et al. (2012a). It should be noted that in the collecting ducts in the outer medulla BGT1 was present in a few sparse cells, presumably interstitial cells (Figure 2B). Also see Figure 7B in Zhou et al. (2012a). Mice lacking BGT1 were viable and, unexpectedly, they concentrated urine and showed no ill effects after 72 h of salt drinking (Lehre et al., 2011; Zhou et al., 2012a). There was no detailed examination of the kidney but, nevertheless, this was in contrast to the severe phenotype of constitutive TonEBP/NFAT5 knockout mice with embryonic/fetal deletion of TonEBP/NFAT5. The few surviving knockout mice displayed renal atrophy, increased apoptosis and impaired activation of osmoprotective genes (Lopez-Rodriguez et al., 2004).

**Figure 3:**
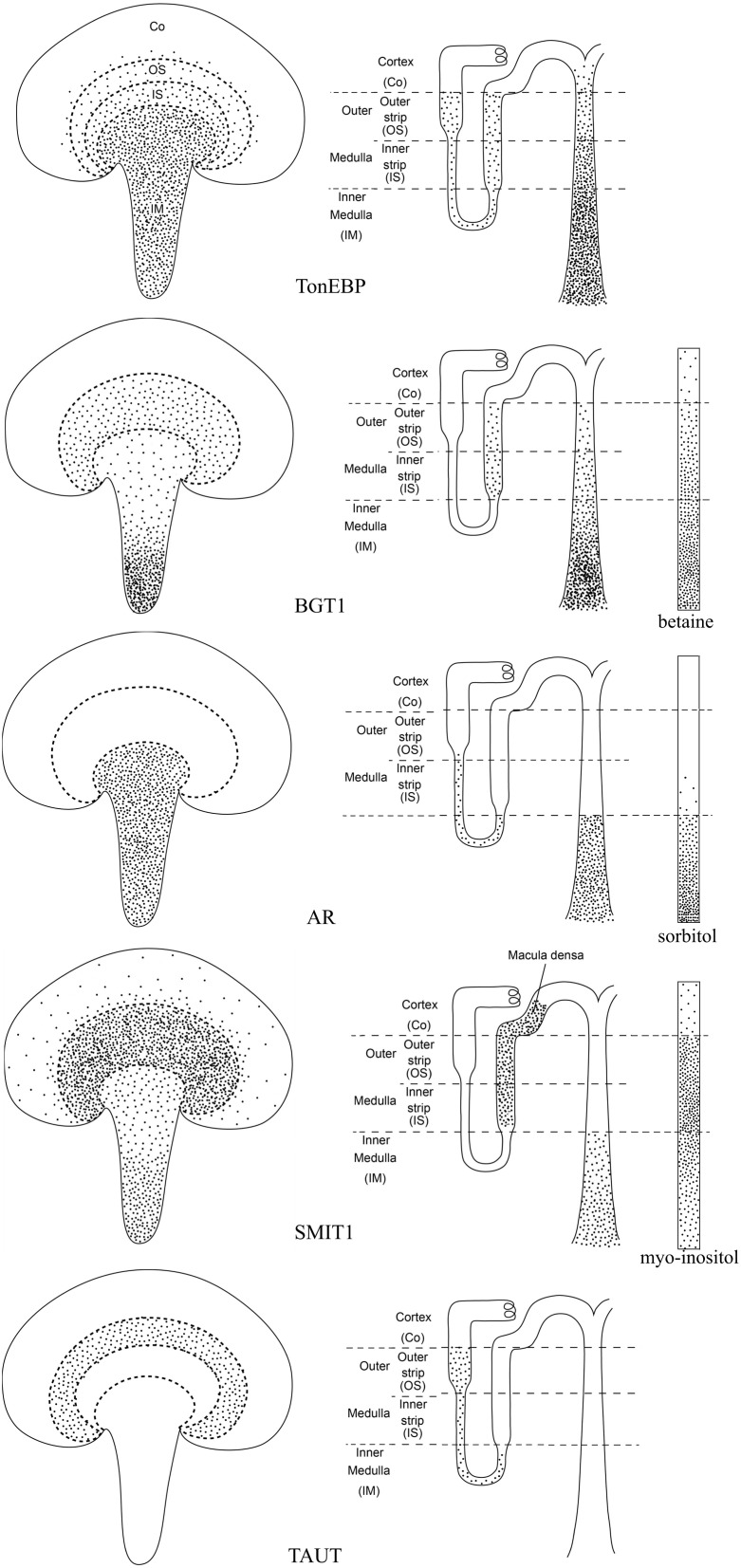
**Schematic illustration of the distributions of TonEBP, BGT1, AR, SMIT1, and TAUT as well as the related osmolytes in the kidney**. TonEBP is present in most tubular profiles in the medulla, including the loop of Henle and medullary collecting ducts and interstitial cells (Han et al., 2004). BGT1 is present in the medullary thick ascending limbs of Henle loop and the medullary collecting ducts (Miyai et al., 1996; Zhou et al., 2012a). Aldose reductase (AR) is in the loop of Henle and inner medullary collecting ducts (Terubayashi et al., 1989; Schwartz et al., 1992; Grunewald et al., 2001). SMIT1 is predominantly present in the medullary and cortical thick ascending limb of Henle's loop and in the cells of the macula densa as well as to a lesser extent in the inner medullary collecting ducts (Yamauchi et al., 1995). TAUT is localized to the proximal tubules in the outer stripe of outer medulla (Park et al., 1989; Lopez-Rodriguez et al., 2004). Organic osmolytes including betaine and sorbitol exhibit their highest concentrations in the papillary tip, except myo-inositol which has similar high concentrations in inner and outer medulla (Wirthensohn et al., 1989; Yancey and Burg, 1989).

TonEBP/NFAT5 is not exclusive for the BGT1 gene, it also regulates several other osmolyte transporter genes including the sodium/myo-inositol cotransporter 1 (SMIT1; slc5a3) (Miyakawa et al., 1999a) and the taurine transporter (TAUT; slc6a6) (Zhang et al., 2003), as well as aldose reductase which converts glucose to sorbitol (Ferraris et al., 1996; Ko et al., 1997). Modulation of these osmolyte cotransporters can be achieved by divergent regulatory pathways (Miyakawa et al., 1999a). The regional distribution of TonEBP/NFAT5, aldose reductase, osmolyte transporters, and osmolytes within the principal zones of the kidney has been described by several different laboratories. These data have been collected from the literature and are summarized in Figure 3. Note the close correlation with the distribution of their respective targets. The widespread distribution of TonEBP throughout the outer and inner medulla allows it to regulate expression of aldose reductase and the osmolyte transporters even though their distributions are different. The most notable differences are the restriction of aldose reductase to the inner medulla and TAUT to the outer stripe of the outer medulla. In contrast, SMIT1 has a widespread distribution in all zones including, due to its location in the macula densa, the cortex. Disruption of the aldose reductase gene caused a defect in urinary concentrating ability and divalent cation homeostasis (Aida et al., 2000). TAUT deficient mice had renal taurine loss and their ability to lower urine osmolality and increase urinary water excretion was impaired (Huang et al., 2006). Mice lacking SMIT1 had more severe defects but death was due to respiratory failure (Chau et al., 2005). Further details of these knockout mice models are summarized in Table 2. Perhaps deletion of two or more of them can reveal the relative importance of these osmolytes in kidney function since betaine, myo-inositol and sorbitol can substitute for each other (Moriyama et al., 1991), consistent with their overlapping distributions (Figure 3). Emerging evidence suggests the involvement of TonEBP/NFAT5 in multiple cellular pathways in addition to organic osmolyte-dependent pathways (Lee et al., 2011), and a review on tonicity-independent regulation of TonEBP/NFAT5 has been published recently (Halterman et al., 2012).

**Table 2:** **Summary of the phenotype of relevant knockout mice**.

**Gene name**	**Expression pattern**	**Phenotype**	**References**
TonEBP (NFAT5, NFATL1, OREBP)	Thymus, placenta, brain, spinal cord, heart, liver > salivary gland, lung, kidney, gut, bladder	Show perinatal lethality; the majority of the few survivors died around P10; display progressive growth retardation; renal atrophy; exhibit abnormal heart development; increased severity of neuronal cell death in ischemic injury; lymphoid hypocellularity and impaired antigen-specific antibody responses; reduced cell proliferation	Trama et al., 2000; Maouyo et al., 2002; Go et al., 2004; Lopez-Rodriguez et al., 2004; Mak et al., 2011, 2012
SMIT1 (SLC5A3)	Kidney > brain	Die shortly after birth probably because of abnormal respiratory rhythmogenesis or severe defects in the pheripheral nerve; no decrease in phophatidylinositol in spite of severe myo-inositol deficiency; lethality can be rescued by supplement of exogenous myo-inositol; shows a lithium-like phenotype but has no effect on lithium-sensitive behavior	Kwon et al., 1992; Berry et al., 2003; Chau et al., 2005; Shaldubina et al., 2006; Bersudsky et al., 2008
Aldose reductase (hAKR1B1, mAkr1b3, EC1.1.1.21)	Testis, heart, retina, len, sciatic nerve, kidney, skeletal muscle, small intestines, thymus, spleen, placenta > brain, lung, pancreas > liver	Viable; fertile; develop polyuria and polydipsia; exhibit a partial defective urine-concentrating ability and a defect in divalent cation homeostasis; leads to nephrogenic diabetes insipidus; ameliorated diabetes-induced renal hypertrophy and nerve degeneration; improves cerebral or retinal ischemic injuries	Nishimura et al., 1988; Cao et al., 1998; Aida et al., 2000; Ho et al., 2000, 2006; Yang et al., 2006; Lo et al., 2007; Zhou et al., 2014
BGT1 (mGAT2, SLC6A12)	Liver > kidney, brain	Viable; fertile; appear to tolerate salt drinking (concentrate urine normally); no decreased susceptibility in electrical and chemical induced seizure	Lehre et al., 2011; Zhou et al., 2012a
TAUT (SLC6A6)	Kidney, brain, retina, small intestine, spleen, heart, skeletal muscle > liver, epididymis, pancreas (islet)	Viable; reduced fertility; reduced weight; decreased taurine levels in a variety of tissues; impaired ability to increase water excretion and to lower urine osmolarity; vision loss due to severe retinal degeneration; show electromyographic abnormalities and reduced total exercise capacity; leads to cardiomyopathy with cardiac atrophy; higher sensitivity to ultraviolet B radiation-induced immunosuppression; loss of ability to self-heal malaria; develop chronic liver disease in old age; reduced ability to develop long-lasting enhancement of synaptic transmission in the striatum; reduced apoptosis of erythrocytes during exposure to osmotic shock or oxidative stress	Liu et al., 1992; Smith et al., 1992; Uchida et al., 1992; Ito et al., 2008; Delic et al., 2010; Zhou et al., 2014

In summary, BGT1 plays an essential role in betaine accumulation by renal medullary cells during adaptation to hypertonic stress. However, the lack of BGT1 and betaine is not critical for survival likely due to compensation by accumulation other osmolytes.

## BGT1 and betaine in liver

**Figure 4:**
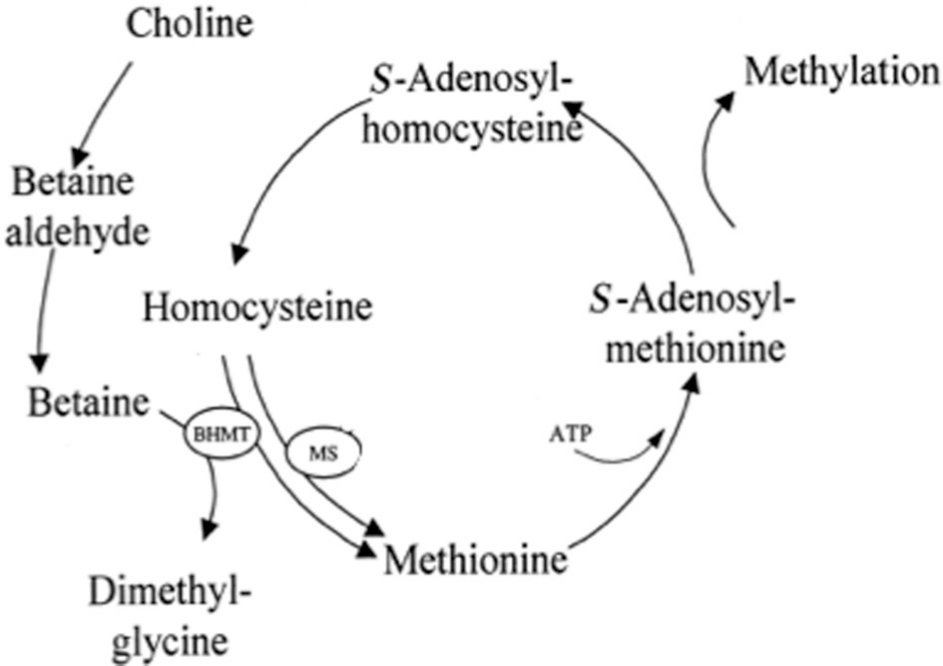
**Role of betaine in the methionine cycle in liver**. Betaine provides an alternative pathway for methylation of homocysteine. BHMT, betaine-homocysteine S-methyltransferase. MS, methionine synthase. Modified from Craig (2004).

Like the kidneys, rat liver also contains high concentrations of betaine (Table 1) (Slow et al., 2009). However, under normal conditions the liver is only slightly hypertonic, approximately 330–335 mosmol/kg (Go et al., 2004), in contrast to kidney medulla where hypertonicity can reach 1000–1200 mosmol/kg (Woo et al., 2000). A role for BGT1 and betaine in liver cell volume regulation cannot be ruled out completely, as suggested by studies on the expression of betaine-homocysteine S-methyltransferase (BHMT1) mRNA. This enzyme removes a methyl group from betaine and is the major betaine catabolizing enzyme in liver (Figure 4). Expression of BHMT1 mRNA in isolated hepatocytes was decreased by hypertonicity consistent with preservation of betaine content for osmoregulation. The reverse occurred during hypotonicity, consistent with removal of intracellular betaine (Hoffmann et al., 2013). In experiments *in vivo*, chronic plasma hyposmolarity in rats was accompanied by decreases in mRNA for TonEBP, SMIT1, and BGT1 in liver (Zhang et al., 2003). Taken together, these findings are a good illustration of the importance of volume regulation for cell survival. The adaptive responses may very well-turn out to be conserved in most cells. In fact, similar adaptive responses have been described in a variety of cell types when grown *in vitro*. Because hepatocytes do not experience osmotic stress under normal conditions *in vivo*, it is unlikely that BGT1 and betaine play important roles in osmoprotection on a daily basis. Thus, it is possible that BGT1 and betaine accumulation may be activated by pathological increases in plasma osmolarity (e.g., hypernatremia or hyperglycemia), but that they play other roles in normal situations.

Another important role of betaine is one-carbon metabolism. This is important for liver function and argues that the primary role of hepatic betaine is to act as a methyl donor. This is in agreement with the finding that the expression of BHMT1 mRNA is at least 50-fold more abundant in mouse liver than any other tissue (Zhou et al., 2012a). A disturbance of hepatic one-carbon metabolism can lead to liver diseases including fatty liver, steatosis and hepatocellular carcinoma (Mato et al., 2008). Betaine contributes significantly to the transmethylation of homocysteine to methionine (Figure 4) because the BHMT1 pathway is a major route for the elimination of homocysteine (cardiovascular risk factor) and catabolism of betaine (Teng et al., 2012a). Deletion of the BHMT1 gene in mice resulted in fatty liver and hepatocellular carcinomas (Teng et al., 2011). Hepatic betaine is derived from two sources: an endogenous source which is *de novo* synthesis from dietary choline (Figure 4), an essential nutrient (Johnson et al., 2010; Zeisel, 2012), and an exogenous source due to dietary betaine (Clow et al., 2008). Which route is the major pathway? In rodent liver betaine is not unusually high (approximately 1–2 μmol/g, Table 1), but when betaine is supplemented in the diet it can increase up to 5 fold (~10 μmol/g) (Schwahn et al., 2004; Clow et al., 2008). Deletion of choline dehydrogenase (converts choline to betaine, Figure 4) in mice did not affect liver function, in spite of alteration of choline metabolites (Johnson et al., 2010). On the other hand, dietary betaine supplements have been reported to reduce liver injury induced by a variety of toxins such as carbon tetrachloride (Junnila et al., 2000), alcohol (Barak et al., 2003; Kharbanda et al., 2012) and lipopolysaccharide (Craig, 2004). Betaine supplements also have been reported to improve liver function in non-alcoholic fatty liver disease (Abdelmalek et al., 2001, 2009; Wang et al., 2010). However, addition of the methyl donor betaine cannot prevent apoptotic death induced by choline deficiency in hepatocytes (Shin et al., 1997). This is probably because part of the choline requirement must be supplied as choline *per se*, but the addition of betaine from diet allows a marked reduction in the total dietary choline requirement (Dilger et al., 2007). To investigate the relative importance of these two sources of betaine (Shin et al., 1997; Dilger et al., 2007; Teng et al., 2012b) identification of the betaine transport mechanisms in hepatocytes will be needed, and a good animal model will be essential.

**Figure 5:**
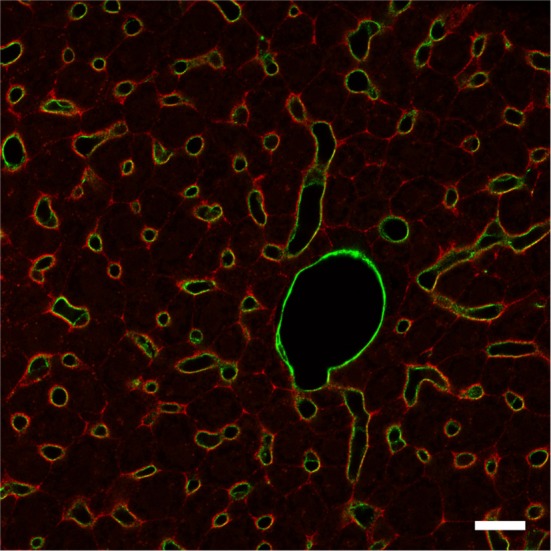
**BGT1 localization in the liver**. The section was double labeled with anti-CD31 antibodies (green; 0.5 μg/ml; endothelial marker) and anti-BGT1 antibodies (red; Ab#594; 1 μg/ml). Scale bars = 20 μm. Immunochemistry was performed using the same materials and procedures described in detail by Zhou et al. (2012a).

The accumulation of betaine has been suggested to be primarily by BGT1 and amino acid transport system A (Craig, 2004; Burg and Ferraris, 2008) but other transporters also may be involved, see below. The contribution of BGT1 to the total capacity of hepatocytes to accumulate betaine has not been determined. Compared to the extensive investigations on renal BGT1, the role of liver BGT1 has received little attention. This in part is because it was thought to be absent from rat hepatocytes, and only present in less abundant Kupffer and endothelial cells (Zhang et al., 1996; Weik et al., 1998). Recently we showed that it is the other way around. BGT1 is primarily expressed in hepatocytes while expression in other liver cell types needs to be tested. BGT1 is present in hepatocyte plasma membranes facing blood vessels (Figure 5) (Zhou et al., 2012a). In the mouse the abundance of BGT1 in liver far exceeds that in the kidney (Zhou et al., 2012a). In contrast to renal medullary cells where BGT1 remains intracellular until the onset of hypertonic stress, BGT1 in hepatocytes is always localized to the plasma membrane even during isotonic conditions (Kempson et al., 2013). This unexpected plasma membrane location in mouse hepatocytes grown in culture under isotonic conditions is probably due to the reported transcriptional activity of TonEBP/NAFT5 at normal osmolarity (Ho, 2003; Zhang et al., 2003; Cheung and Ko, 2013) and the differential expression of BGT1 mRNA isoforms in liver (Takenaka et al., 1995). However, it has not been determined if liver BGT1 is regulated by TonEBP. Thus, liver BGT1 is ideally localized for import of betaine into hepatocytes from the circulation which would maintain the supply of betaine for hepatocyte metabolism. It also has been reported (Wettstein et al., 1998) that hepatocyte uptake of betaine was not stimulated by hyperosmolarity, and it was suggested that hepatocytes may release some of the accumulated betaine for use as an osmolyte by nearby Kupffer cells and endothelial cells. Kupffer cells, the resident liver macrophages, adhere to the endothelial cells of the liver sinusoids and account for the central role of the liver in systemic and regional defense. They are the first macrophage population in the body to encounter bacteria and endotoxins that are delivered to the liver from the gastrointestinal tract via the portal vein (Bilzer et al., 2006). In this location the capacity for osmoadaptation may be critical for the survival and normal function of Kupffer cells.

BGT1 transport activity in hepatocytes in primary cell culture was characterized using betaine as the substrate rather than GABA because of the report of a GABA carrier (GAT2) in hepatocytes (Ikeda et al., 2012). In our initial experiments the uptake of [^14^C]betaine by isolated hepatocytes was sodium dependent and was partly inhibited by quinidine and nipecotic acid, similar to the activity of the renal BGT1 transporter (Yamauchi et al., 1992; Kempson et al., 2003). We also noted that [^14^C]betaine transport was partly inhibited by α-methylaminoisobutryic acid and carnitine (Kempson et al., 2013). This implies that multiple pathways might be involved in accumulating betaine, such as the system A amino acid transporter family (SNAT 1, 2 and 4) (Hatanaka et al., 2001; Bode et al., 2002) and the ubiquitous carnitine transporter (OCTN2) (Yokogawa et al., 1999). If true, this would help explain why BGT1 knockout mice are not severely impaired (Lehre et al., 2011). Determining the contribution by BGT1 to the total betaine uptake will require identification of the betaine transport mechanisms in hepatocytes from the BGT1 knockout mice.

## Conclusions

Betaine is found in many plants, animals, microorganisms and in mammals. In addition to its role as an osmolyte, its metabolic role has been shown to be important in protection of the liver and other tissues and in alleviating cardiovascular risk factors such as homocysteine. Consequently it has been suggested that betaine is an important nutrient for prevention of chronic disease (Craig, 2004). A summary of the multiple roles of betaine and BGT1 in the tissues discussed in this review is presented in Table 3. Both are present in brain but their roles are least understood in this tissue. Betaine is of undoubted importance for liver metabolism, but the importance of BGT1 for betaine transport remains unclear even though its abundance in liver is almost 50-fold greater compared to the kidney. The best documented roles are in the kidney where betaine serves as one of several compatible osmolytes that are accumulated by cells in the renal medulla in order to adapt to the high extracellular osmolarity. This is facilitated by upregulation of BGT1 expression mediated by the transcription factor TonEBP/NFAT5. Due to the significant difficulties in replicating *in vitro* the normal cellular microenvironments present *in vivo*, complete evaluation of the roles of BGT1 and betaine (and other osmolyte transporters) in these tissues *in vivo* will require further genetic manipulation in whole animals.

**Table 3:** **Summary of functions of BGT1 and betaine in mouse tissues**.

**Tissue**	**Primary substrate for BGT1**	**BGT1 abundance in tissue**	**BGT1 location**	**Principal role of BGT1**	**BGT1 regulated by TonEBP**	**Betaine content of tissue**	**Principal role of betaine**
Brain	Unknown (Betaine or GABA)	Low	Leptomeninges	Unknown. Low affinity for GABA compared to GAT transporters	Unknown	Low	Not an important osmolyte. Possible role as extracellular signaling ligand
Kidney	Betaine	High	Basolateral in medullary cells (thick ascending limbs of Henle and collecting ducts). Highest levels at the tip of the renal papilla	Betaine transport for cell volume regulation during hypertonic stress	Yes	High	Compatible osmolyte in medullary cells
Liver	Betaine	Highest	Hepatocyte plasma membranes	Primary role in betaine transport for one-carbon metabolism. Possible secondary role in volume regulation	Unknown	High	Primary role as methyl group donor in liver metabolism (methionine cycle). Possible secondary role as osmolyte

*See text for further details*.

The knockout mouse models currently available (Table 2) also serve as excellent negative controls for verifying antibody specificity which is essential for accurate interpretation of immunohistochemical data (Danbolt et al., 1998; Holmseth et al., 2005; Holmseth et al., 2012). The story about EF1502 and inhibition of brain BGT1 illustrates how much the biomedical research community relies on accurate localization data to interpret data obtained with other methods such as pharmacology experiments (Lehre and Rusakov, 2002; Rusakov et al., 2005; Holmseth et al., 2012). When designing new CNS active drugs virtually all receptors, metabolic enzymes and transporters should be considered as valid targets (Kowalczyk et al., 2012; Salat et al., 2012). So although EF1502 was tested on a fair number of targets, it is not possible to test all. Further, it is important to keep in mind that cultured astrocytes may differ substantially from mature brain astrocytes (Cahoy et al., 2008).

The ability to use osmolytes to adapt to osmotic stress has been conserved in all cells because it is vital for survival and TonEBP/NFAT5 is expressed at basal levels in all tissues in the body (Halterman et al., 2012). However, the many observations of osmolyte accumulation by cultured cells during hypertonic stress *in vitro* should not lead to the conclusion that the same cells are exposed to hypertonic stress *in vivo*. In fact, only a limited number of tissues experience hyperosmotic stress under normal conditions. It has been suggested that the activation of TonEBP/NFAT5 by hypertonicity in the kidney medulla may be a specialized adaptation of its normal role under iso-osmotic conditions (Trama et al., 2002), namely its regulation by tonicity-independent factors critical for cell survival, mitogenesis and migration (Halterman et al., 2012). The presence of TonEBP/NFAT5 in all cells allows the possibility of regulation by cell-specific factors and permits a role in activating cell-specific gene transcription (Cheung and Ko, 2013).

Finally it should be noted that the responses to hyperosmotic stress have been thoroughly investigated and identified in the kidney due to its unique structure and function. More recently, studies have revealed that non-renal tissues commonly experience hyperosmotic stress, especially under pathological conditions such as hypernatremia and hyperglycemia (Brocker et al., 2012). For example, in response to the chronic hyperglycemia found in diabetics, glucose flux through the polyol pathway is increased and accounts for about one third of total glucose consumption. In this pathway aldose reductase converts glucose to sorbitol and the resultant accumulation of intracellular sorbitol causes hyperosmotic stress (Cheng and Gonzalez, 1986). Aldose reductase occurs not only in the renal medulla but also in other organs and has long been believed to be responsible for secondary diabetic complications such as retinopathy, neuropathy, nephropathy, and caractogenesis.

Taking a broader view, a number of studies have reported a strong association between localized hypertonicity and inflammation (Hubert et al., 2004; Schwartz et al., 2009). The contribution of osmotic stress to the development and progression of chronic inflammatory diseases, such as arthritis and inflammatory bowel disease (IBD), is not well-understood. However, in the case of IBD, recent research suggests that episodes of local hyperosmotic stress can upregulate the release of pro-inflammatory cytokines by colorectal epithelial cells (Abolhassani et al., 2008; Schwartz et al., 2008). Future studies may reveal that therapeutics targeting localized hyperosmotic adaptation could represent a novel class of drugs for the treatment of many disorders. For example, inhibitors of aldose reductase have been shown to prevent inflammatory complications such as sepsis and asthma (Srivastava et al., 2005; Ramana, 2011). Perhaps localized inhibition of other features of hyperosmotic adaptation, such as BGT1 and other osmolyte transporters, also may be useful in preventing inflammation.

## Author contributions

All of the authors made significant contributions to this review, reviewed several drafts prior to submission, and approved the final version. Yun Zhou and Niels C. Danbolt were primarily responsible for writing the section on the brain. They also contributed to the section on the liver, provided Figures 1–3, 5 and Tables 2, 3. Stephen A. Kempson was primarily responsible for writing the section on the kidney, contributed to the section on the liver, provided Figure 4 and Table 1, and prepared the final version.

### Conflict of interest statement

The authors declare that the research was conducted in the absence of any commercial or financial relationships that could be construed as a potential conflict of interest.

## Abbreviations

BGT1: sodium/chloride-dependent betaine-GABA transporter (slc6a12)
BHMT1: betaine:homocysteine –S-methyltransferase
EF1502: *N*-[4,4-bis(3-methyl-2-thienyl)-3-butenyl]-4-(methylamino-4,5,6,7-tetrahydrobenzo[*d*]isoxazol-3-ol
GABA: γ-aminobutyric acid
GAT: GABA transporter
IBD: inflammatory bowel disease
MDCK: Madin-Darby canine kidney
MTHFR: methylenetetrahydrofolate reductase
OCTN2: carnitine transporter (Slc22a5)
SMIT1: sodium-dependent myo-inositol cotransporter-1 (slc5a3)
SNAT1: Slc38a1 (previously referred to as ATA1, GlnT, SA2, SAT1, or mouse NAT2)
SNAT2: Slc38a2 (previously referred to as SAT2, ATA2, KIAA1382, SA1)
SNAT4: Slc38a4 (previously referred to as PAAT, NAT3, ATA3, SAT3), amino acid system A transporters
TAUT: sodium/chloride-dependent taurine transporter (slc6a6)
TonEBP: tonicity-responsive enhancer binding protein, also known as NFAT5, nuclear factor of activated T cells-5.
